# Supplementary figures and images for: Characterization of the endometrial, cervicovaginal and anorectal microbiota in post-menopausal women with endometrioid and serous endometrial cancers
Source: PLoS One. 2021 Nov 5;16(11):e0259188. doi: 10.1371/journal.pone.0259188 (PMC8570463; doi:10.1371/journal.pone.0259188)

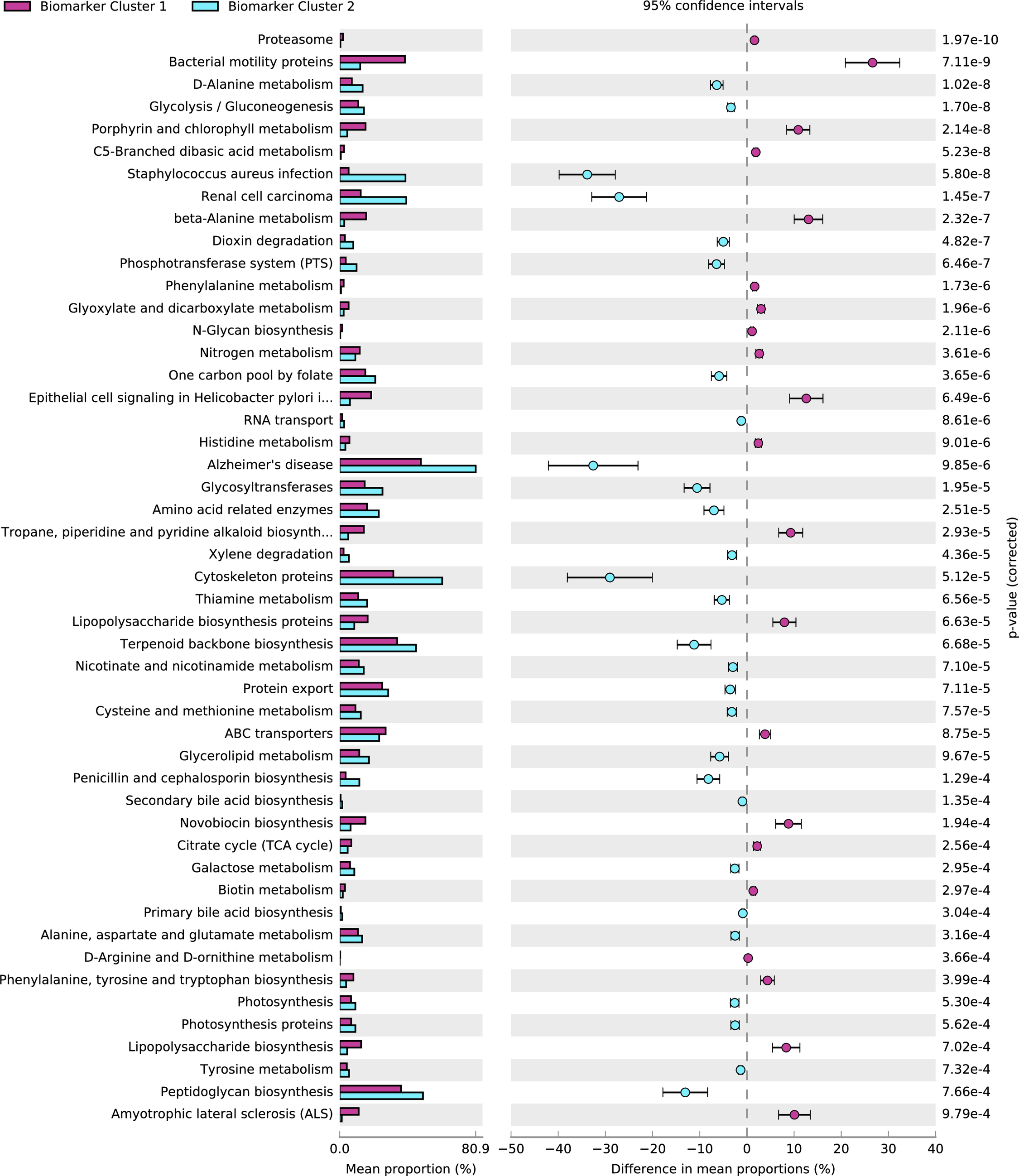

Supplement: S1 Fig — (TIF) [file pone.0259188.s001.tif]
